# Post-acute sequelae of COVID-19 and the mental health implications

**DOI:** 10.15190/d.2021.19

**Published:** 2021-12-31

**Authors:** Namrata Walia, Jessica Olivia Lat, Rabeet Tariq, Surbhi Tyagi, Adam Manzoor Qazi, Syeda Wajiha Salari, Amina Jafar, Tasneem Kousar, Sherrie Bieniek

**Affiliations:** ^1^Baylor College of Medicine, Houston, Texas, United States; ^2^Ateneo School of Medicine and Public Health, Philippines; ^3^Liaquat National Hospital and Medical College, Karachi, Pakistan; ^4^International School of Medicine, Jinan University, Guangzhou, China; ^5^The Hospital for Sick Children, Toronto, Canada; ^6^Rawalpindi Medical University, Pakistan; ^7^King Edward Medical University, Pakistan; ^8^Foundation University Medical College, Rawalpindi, Pakistan; ^9^Larkin Community Hospital, South Miami, Florida, United States

**Keywords:** COVID-19, long-haulers, long COVID-19, multi-organ, mental health.

## Abstract

Post-acute sequelae of COVID-19 (PASC) or more commonly known as Long COVID-19, is the term given to persistent symptoms 12 weeks from the initial presentation of COVID-19 infection. Several multi-organ symptoms have been reported by patients. Some common symptoms include headaches, fatigue, memory impairment and mental health complications such as anxiety and depression. People with previous psychiatric diagnosis are at greater risk of developing longer mental health implications from persistent COVID-19 symptoms. Additionally, healthcare workers are at increased risk of being long haulers leading to burnout and exhaustion. The objective of this review article is to provide comprehensive evidence from existing literature on various symptoms reported by patients experiencing Long COVID-19 and the rate of occurrence of such symptoms in different populations. A long-term disease surveillance is required to further understand the persistent symptoms or the long-term impact of this infection.

## SUMMARY

1. Introduction

- 1.1 What is Long COVID-19?

- 1.2 Overview of symptoms

*2. Epidemiology*

- 2.1 Definition and Classification

- 2.2 Prevalence of symptoms

- *2.3 *Implications on healthcare workers

- 2.4 Who is at risk?

*3. Mental health implications*

- 3.1 Neuro-psychiatric symptoms

4. Conclusion

## 1. Introduction

The pathogen responsible for the COVID-19 pandemic is severe acute respiratory syndrome coronavirus 2 (SARS-CoV-2). Like the previous coronavirus, SARS-CoV-1, this novel coronavirus can result in severe lower respiratory tract infection, often complicated by acute respiratory distress syndrome (ARDS)^1^. SARS-CoV-2, however, can also present with a broader variability in clinical syndromes and severity. Some of the most common symptoms of mild to moderate infection are cough, fever, and fatigue, where cough and fever are the most predominant. SARS-CoV-2 infection can also present with diarrhea, rhinorrhea, sore throat, myalgia, sinus congestion, loss of sense of smell or taste, muscle aches, and headaches^1^. However, approximately 40-45% of patients are asymptomatic during the duration of the infection, but they can transmit the virus to others for an extended period of time^2^. The main symptoms of COVID-19 infection usually appear within 2 to 14 days post-exposure. Duration of symptoms varies per individual, but most people recover by two weeks while some take longer to recover from COVID-19. Since the onset of the pandemic, approximately 5% of the COVID-19 cases were admitted to the hospital, of which approximately 75% recovered. The hospitalization of COVID-19 has been on a steady decline in the United States since January 11, 2021, with a drop in hospitalization by approximately 73% between the 2nd week of January 2021 and the 2nd week of May 2021^3^.

### 1.1 What is Long COVID-19?

The global COVID-19 pandemic has caused grievous mortality, morbidity, and worldwide disruption. The Chinese Center for Disease Control and Prevention (CDC) in February 2020 published a record of 72314 cases of SARS- CoV-2 patients from December 8, 2019 to February 20, 2020, among which 36160 cases (81%) had mild disease, 6168 cases (14%) had severe disease, and 2087 cases (5%) were critical^4-6^. A cohort study on the risk of mortality in patients infected with SARS-CoV-2 variant of concerns (B.1.1.7) showed an increased risk of death (central estimate 64%) than equal individuals with previously circulating cases^7^. Greenhalgh et al. published that some of the affected individuals have reported persistent symptoms or develop new ones after 3 weeks from the onset of the first symptom and beyond 12 weeks^8-10^. The symptoms include fatigue, mental fog, inability to do routine activities, anxiety, depression, tachycardia, and dyspnea^11^. A study from Italy showed that 87% of recovered individuals showed one persistent symptom, including a high proportion of individuals who still had fatigue (53.1%), dyspnea (43.4%), joint pain (27.3%), and chest pain (21.7%) even after 60 days of recovery^5,12^. The illness was called “Long COVID-19,” first used by Elisa Perego on Twitter on May 20, 2020, who summarized the illness as cyclical, multiphasic, and progressive^4,5^. An article published by Yong and Watson mentioned that SARS-CoV-2 infection could last for months and featured nine people who called themselves “long haulers”^13^. Other related terms specific to the disease are “Post COVID-19 Syndrome” or “Post-acute COVID-19” as persistent symptoms or new symptoms are seen after 3 weeks of SARS-CoV-2 infection, and “Chronic COVID-19” refers to symptoms seen after 12 weeks of SARS-CoV-2 infection^5,14^. In February 2021, National Institutes of Health (NIH) director Francis Collins termed the effects of COVID-19 after the acute phase as “Post–Acute Sequelae of COVID-19” (PASC) and announced an initiative of $ 1.15 billion over four years to identify the cause and means of prevention and treatment of affected individuals recover even after weeks or months of SARS-CoV-2 infection^15^. The initiative aims to address the clinical spectrum of recovery from acute SARS-CoV-2 infection over time to study the incidence, prevalence, natural history (including duration), distinct phenotypes of infected individuals with other sequelae, and impact of treatment of acute SARS-CoV-2. The initiative also focuses on the pathogenesis of conditions that develop over time, resulting in organ dysfunction or predisposing the infected individuals to other disorders. NIH issued two research opportunities for the PASC initiative. The first research opportunity focuses on three research areas: Clinical Recovery Cohort studies, Autopsy Cohort studies, and EHR with other data-based studies. The second research opportunity focuses on Clinical Science Core, the Data Resource core, and PASC biorepository^15-17^.Several new studies have reported prevailing symptoms and quality of life-related health issues in patients recovering from COVID-19. A recently published meta-analysis^18^ reported that the prevalence of some of the common symptoms of PASC reported in literature include fatigue (64%), cough (22.5%), dyspnea (39.5%), anosmia (20%), arthralgia (24.3%), chest pain (10%), headache (21%), sleep disturbances (47%), and mental health problems (14.5%). The authors reported a pooled prevalence of symptoms including pain (42%, 95% CI: 28%–55%) and anxiety/depression (38%, 95% CI: 19%–58%)^18^.

### 1.2 Overview of symptoms

Post-acute sequelae of COVID-19 is a wide term that includes a diverse variety of physical and psychiatric problems persisting weeks or months after COVID-19 infection. These need to be addressed more frequently so that they can be treated with evidence-based guidelines. The most prevalent consequences in post COVID-19 period are fatigue, early exhaustion, stress, brain fog (memory and attention deficit), palpitations, and dyspnea. There are several other symptoms reported which are less common but affect the social and personal life of COVID-19 recovered patients in various ways. Some of these problems include psychiatric issues, for example, depression, anxiety, disturbed sleep patterns, mood changes and headache, life-threatening cardiovascular problems (tachycardia, chest pain, venous thromboembolism, stress cardiomyopathy), gastrointestinal disturbances including stress-induced anosmia and ageusia, cough, myalgias and arthralgias limiting daily activities, dermatological symptoms like skin rashes or hair loss creating cosmetic concerns and hormonal responses like diabetic ketoacidosis in diabetic patients, or thyroiditis etc. These multisystem effects can sometimes prove hazardous for patients in post COVID-19 period. They may survive through COVID-19 infection, but post-acute sequelae of COVID-19 can be life-threatening for them.

## 2. Epidemiology

### 2.1 Definition and Classification

Several terminologies have been used to define sequelae of COVID-19, including Post-acute COVID-19 syndromes^19^, Long COVID-19^4,20^, and Chronic COVID-19 Syndromes^20^. Al-Jahdhami et al. used the terms including Post-acute COVID-19 syndromes and Long COVID-19 interchangeably^9^. National Institute of Health Care and Excellence (NICE) guidelines classify acute COVID-19 (symptoms for up to 4 weeks), ongoing symptomatic COVID-19 (from 4 to 12 weeks), and post COVID-19 (more than 12 weeks)^21^. Venkatesan's commentary of NICE guidelines also uses the terms interchangeably and defines them as an “ongoing symptomatic COVID-19 for people who still have symptoms between 4 and 12 weeks after the start of acute symptoms”; and post COVID-19 syndrome for people who still have symptoms for more than 12 weeks after the start of acute symptoms^22^. Greenhalgh et al. define post-acute COVID-19 as extending beyond three weeks from the onset of first symptoms and chronic COVID-19 symptoms extending beyond 12 weeks^10^. Nalbandian et al. define post-acute COVID-19 syndrome as persistent symptoms or long-term complications of SARS-CoV-2 infection more than four weeks from the onset of symptoms^19^. Datta et al. proposed that the acute infection of COVID-19 lasts two weeks, followed by Post-Acute Hyperinflammatory Illness and, lastly, Late COVID-19 sequelae after four weeks^23^. Fernández-de-las-Peñas et al. classified the symptoms in 4 phases: Transition Phase (up to 4–5 weeks); Phase 1: Acute post COVID-19 symptoms (5th -12th week); Phase 2: Long post COVID-19 symptoms (12th -24th week); Phase 3: Persistent post COVID-19 symptoms (symptoms lasting more than 24 weeks)^24^.

### 2.2 Prevalence of symptoms

Carfì et al. studied 143 patients after their recovery from COVID-19. 12.6% of the cases were completely free of any COVID-19–related symptom, 87.4% reported persistence of at least 1 symptom, among which 32% had 1 or 2 symptoms, and 55% had three or more symptoms, the most common ones being fatigue (53.1%), dyspnea (43.4%), joint pain, (27.3%) and chest pain (21.7%)^12^. Garrigues et al. assessed patients more than 100 days after their discharge via phone calls and reported that the most common persistent symptoms were fatigue (55%), dyspnea (42%), loss of memory (34%), concentration disorders (28%) and sleep disorders (30.8%)^25^. Chopra et al. conducted an observational cohort study on 1648 patients for 60 days, out of whom 488 (41.8%) completed the 60-day post-discharge survey. There were 159 patients who reported cardiopulmonary symptoms like cough and dyspnea, 65 reported loss of taste or smell, 58 reported new or worsening difficulty in completing activities of daily living, and 238 reported being emotionally affected by their health^26^. Carvalho-Schneider et al. followed recovered COVID-19 patients at day 30 and 60 and reported the most common symptoms at disease onset were flu-like symptoms (87%), anosmia/ageusia (59%), and fever (51%^27^^27^^27^. Several studies also reported an increased risk of venous thromboembolism^27-29^ and bleeding^29^.There is also a risk of acute and post-acute neurological complications of COVID-19^30^. Mao et al. conducted the first retrospective, observational case series on neurologic symptoms of COVID-19 patients (n=214) and reported 36.4% had neurologic manifestations. Manifestations such as acute cerebrovascular disease and conscious disturbance were more likely to develop in patients with severe infection. However, some patients presented to the hospital with only neurologic manifestations without typical symptoms^31^. Ortelli et al. reported post COVID-19 neuromuscular fatigue, cognitive fatigue, apathy, and executive dysfunction^32^. Varatharaj et al. developed online case report notification portals and reported 153 cases, out of which 125 patients had confirmed COVID-19 infection. 65% presented with the broad clinical syndrome of a cerebrovascular event (74% had an ischemic stroke, and 12% had an intracerebral hemorrhage). 31% presented with altered mental status (9 with unspecified encephalopathy, 7 with encephalitis). 59% of patients with psychiatric disorders (43% had new-onset psychosis, 26% had a neurocognitive syndrome, and 30% had other psychiatric disorders)^33^. Paterson et al. classified the neurologic symptoms into 1-encephalopathies (delirium/psychosis and no distinct MRI or CSF abnormalities); 2-inflammatory CNS syndromes (including encephalitis, encephalomyelitis, myelitis); 3-Ischaemic strokes; 4-peripheral neurological disorders (Guillain-Barré syndrome, brachial plexopathy) and 5-miscellaneous central disorders which did not fit the other categories^34^. Other nervous manifestations include headaches^35^ and seizures^36,37^.

### 2.3 Implications on healthcare workers

Amidst this pandemic, healthcare workers worldwide have been front liners in the fight against COVID-19. Although the media, politicians, and the general population have massively praised the heroic efforts of healthcare workers, they have been impacted by the pandemic in more ways than the general population, and its consequences can have a lasting impact. While most other professionals started working from home to protect themselves and their families from this virus, the healthcare workers are required to continue coming to their workplace^38^. In general, the frontline medical staff is at an increased risk of mental health problems compared with the general population. This coupled with the psychological strain of dealing with the unprecedented nature of this pandemic, a tremendously increased workload, fear of contracting the infection and then spreading it to their near and dear ones, and an inability to give their best at work due to limitations caused by the symptoms of Long COVID-19/PASC further amplifies the risk of burnout, stress, depression and mental trauma and suicidal ideation among frontline workers^39^. These ‘Long Haulers’ are more likely to develop Post traumatic stress disorder (PTSD) after seeing so many patients on a daily basis, fighting for their lives, and then finally losing the battle against this pandemic. For those with a genetic predisposition towards conditions like obsessive compulsive disorder (OCD), the stress of the pandemic is likely to trigger or further worsen their OCD. Similarly, people who tend to get anxious easily are likely to experience a worsening of their anxiety. A survey of 1,257 healthcare workers attending to COVID-19 patients in China showed that 83 out of 200 of these individuals had worsening depressive symptoms, anxiety and general distress when compared with peers who were not directly working with COVID-19 patients. The study also found that individuals who had a strong social and support network were less likely to suffer from these mental health implications of Long COVID-19^40^. However, despite the gravity of this situation, many healthcare workers have not been seeking help for these mental issues either due to lack of psychological support, fear of being judged by their peers due to stigma associated with psychological problems, limited resources, lack of information about available mental health support or time constraints^41^.

### 2.4 Who is at risk?

The CDC suggests that older patients and those with underlying health conditions may be at more risk of experiencing PASC compared to healthy adults, but further investigation is needed to understand this condition^42^. In contrast, Sudre et al. found the incidence of Long COVID-19 to be higher in the individuals aged 18–49 with greater risk in increasing age, increased body mass index and female sex. Having more than five symptoms during the first week of acute disease was also found to be a predictor of Long COVID-19^43^. Huang et al. and Jacobs et al. found that older patients experienced more PASC symptoms. Several studies have also shown that women more frequently report PASC symptoms as early as four weeks up to 6 months after recovery from COVID-19^44-48^. There is also an association between persistent PASC symptoms and patients who required hospital admission, but studies show conflicting results. According to Huang et al., Halpin et al., and Taquet et al., a direct correlation is seen between increasing severity of COVID-19 illness and reporting more PASC symptoms. They found that ICU-admitted patients who required invasive ventilation were more likely to report physical and mental problems weeks after recovery from COVID-19^44,47,49^. However, a study conducted in France that followed patients 110 days after discharge showed that while hospital-admitted patients experienced persistent fatigue, loss of memory and concentration, and sleep disorders, there was no significant difference between ward-admitted and ICU-admitted patients^25^. A different study conducted in Italy surveyed patients one month after hospital discharge and noted an inverse relationship between duration of hospitalization and PASC symptoms^46^. Patients managed at home for mild COVID-19 reported feeling more anxious and depressed, suggesting that less healthcare support perceived by patients contributed to isolation and loneliness during recovery. Mazza et al. also analyzed that woman, patients who managed symptoms at home, and patients with prior psychiatric history have higher self-reported symptoms of PTSD, depression, anxiety, insomnia, and obsessive-compulsive disorder. In addition, Taquet et al. reported that patients with prior psychiatric history contributed to psychological distress after recovering from COVID-19. They surveyed patients 14 to 90 days after hospital discharge and found that there is twice the risk of being diagnosed with a psychiatric disorder for the first time. The most common diagnoses were anxiety disorders, insomnia, and dementia^49^. In contrast, Janiri et al. found no established association with a history of psychiatric disorders that would influence psychological distress after diagnosis. Instead, their study showed that patients who exhibit cyclothymic and depressive temperaments before COVID-19 infection were more likely to develop psychological distress after recovery^48^.

## 3. Mental health implication

In addition to the debilitating physical symptoms of COVID-19 infection, the prolonged sequelae of events in people with PASC/ Long-COVID-19 has resulted in various psychiatric issues, including depression, sleep difficulties, mild to severe anxiety, PTSD, phobias, avoidant behavior, obsessive compulsive symptoms, and in rare cases, dementia in the elderly population. People with prior history of psychiatric illness are at greater risk for mental health implications^19^. Alcohol and other substance use have reportedly increased in long haulers as a coping strategy from stress caused by the long period of illness and other psychosocial causes. In a meta-analysis, the post COVID-19 psychiatric symptoms reported were depressed mood, insomnia, anxiety, irritability, memory impairment, fatigue, delirium, agitation, and altered level of consciousness. The prevalence of post-traumatic stress disorder was reported to be 32.2%, depression 14.9%, and anxiety disorders 14.8%^50^. Rossi Ferrario et al. reported the most common post COVID-19 psychological issues were acute stress disorders (18.6%), anxious and demoralization symptoms (26.7%), depression (10.5%), and troublesome grief (8.1%)^51^.Loneliness, economic losses/loss of job, and increased responsibility towards children and other family members due to closure of daycares/schools/workplace and general lack of social support during these tough times further exacerbate these adverse mental health effects^52^. On one hand, social distancing and quarantine help limit the spread of this virus, but on the other hand, it has led to a sense of "lack of meaning to life," a constant fear of losing someone to this deadly virus, and an increased incidence of substance use which in turn has resulted in a growing number of cases of child/elderly abuse and domestic violence^53^. These place an immense medical, economic, and psychological burden on people, especially the low-income tier, those who are underinsured or not insured, undocumented immigrants, the homeless, and other marginalized groups^52^. A study on COVID-19 patients discharged from hospitals showed that 10% of patients had developed anxiety symptoms, 19% had depressive symptoms, and 12% developed PTSD, thus revealing the gravity of mental health implications of COVID-19 pandemic^54^.

### 3.1 Neuro-psychiatric symptoms

The long-term complication in people who suffered early neurological symptoms due to COVID-19 infection such as stroke, are disabilities requiring rehabilitation. However, the most commonly reported neurologic symptoms include headache, vertigo, loss or lack of taste and smell, memory impairment, and inability to concentrate (brain fog). The most unusual, reported symptom is called “brain fog'', which was reported as a long-term symptom of COVID-19 by the Centre of Disease Control and Prevention (CDC). Clinically known as dysexecutive syndrome, it is defined as a state of confusion and mental sluggishness. It often presents after recovery as a persistent cognitive sluggishness. Graham et al. published a prospective study of 100 patients at NeuroCOVID-19 clinic of Northwestern Memorial Hospital, Chicago. The most common neurological symptom reported by patients (81%) was a non- specific cognitive issue mentioned as "brain fog"^55^. According to pathologists at Johns Hopkins University in Baltimore and Brigham and Women's Hospital in Boston, “large bone marrow cells called megakaryocytes may be responsible for this presentation^56,57^. These megakaryocytes migrate to the brain triggered by activity of SARS-COV-2. They block blood flow through capillaries in the cerebral cortex and lead to a state of focal neurological impairment. Stefano et al. proposed that SARS-CoV-2 can damage the mitochondrial energy metabolism and it results from integration of the viral genome into the host cell mitochondrial matrix. Mitochondrial dysfunction with a pro- inflammatory response contributes to neuronal dysfunction and results in "brain fog”^58^.

## 4. Conclusions

Given the recent emergence of the incidences of Long COVID-19 symptoms, it is unclear how long it can take for the symptoms to completely resolve. A long-term disease surveillance is required to understand the persistent symptoms or the long-term impact of this infection. However, some researchers have suggested holistic and evidence-based guidelines for the management of symptoms of long haulers. Providing additional support through long-term follow ups and disseminating resources on self-management are warranted.

## KEY POINTS

*◊Post-acute sequelae of COVID-19 (Long COVID-19) presents with multi-organ symptoms, such as headaches, fatigue, memory impairment and mental health complications*.

*◊A long-term disease surveillance is required to understand the persistent symptoms or the long-term impact of this infection*.

## OPEN QUESTIONS

*◊* *Will the presenting symptoms change with the new virus variants?*

*◊* *What are the long-term mental health and neurological implications of COVID-19?*
